# Socio-Demographic Indicators, Intelligence, and Locus of Control as Predictors of Adult Financial Well-Being

**DOI:** 10.3390/jintelligence5020011

**Published:** 2017-04-06

**Authors:** Adrian Furnham, Helen Cheng

**Affiliations:** 1 Department of Psychology, University College London, London WC1E 6BT, UK; h.cheng@ucl.ac.uk; 2 BI Norwegian Business School, Nydalsveien 37, 0484 Oslo, Norway; 3 ESRC Centre for Learning and Life Chances in Knowledge Economies and Societies, Institute of Education, University College London, London WC1H 0AL, UK

**Keywords:** financial well-being, intelligence, locus of control, malaise, education and occupation, longitudinal

## Abstract

The current study investigated a longitudinal data set of 4790 adults examining a set of socio-demographic and psychological factors that influence adult financial well-being. Parental social status (at birth), childhood intelligence and self-esteem (at age 10), locus of control (at age 16), psychological distress (age 30), educational qualifications (age 34), current occupation, weekly net income, house ownership status, and number of rooms (all measured at age 38 years) were examined. Structural Equation Modelling showed that childhood intelligence, locus of control, education and occupation were all independent predictors of adult financial well-being for both men and women. Parental social status and psychological distress were also significant predictors of the outcome variable for men, but not for women. Whereas for women, in comparison to men, the effects of current occupation and childhood intelligence on the outcome variable appeared to be stronger. The strongest predictor of adult financial well-being was current occupational prestige, followed by educational achievement. The gender deferential of financial well-being indicators and its implications are discussed.

## 1. Introduction

Financial well-being as a research topic has drawn interests not only for financial planners, economists, sociologists, and policy makers, but also for psychologists. The different disciplines emphasise different factors that lead to financial status in adulthood. This study aims to investigate a set of socio-demographic factors (parental social status, education and occupation) as well as psychological factors (intelligence, self-esteem, locus of control, malaise) that influence adult financial well-being indicated by weekly net income, house ownership status, and living space (number of rooms). The study had a large, nationally representative sample in the UK, and had a particular focus on psychological factors (intelligence, locus of control, malaise) and the extent to which they independently predict adult financial well-being.

Previous studies have established findings on the links between family background, childhood intelligence and later educational and occupational outcomes [1,2,3,4,5,6,7,8,9,10,11,12], between childhood cognitive development and adulthood socioeconomic status and mental health [13,14] using a large American sample showed that each IQ test point raises income by between $234 and $616 per year after holding numerous other factors constant.

However, few studies have examined the effects of locus of control, intelligence, and mental health on adult financial well-being. Many studies have demonstrated the predictive power of locus of control with respect to many life outcome variables including health and financial well-being [15,16,17] showed locus of control, measured at age 10 years predicted social class at age 30 years. Locus of control has been linked to attitudes and behaviour with respect to money [18,19]. Those with internal locus of control tend to be more strongly motivated to exercise plan and take responsibility for their actions.

There is also a literature on personality and occupational and financial success which suggests particular personality variables, namely Conscientiousness positively and Neuroticism negatively are related to career success [20,21]. In this study we examine the effect of early adult Malaise (a strong correlate of Neuroticism) on mid adult financial success. Longitudinal studies have shown that early indicators of instability/malaise are negatively linked to educational and occupational outcomes [17]. We also examined self-esteem measured at aged 10 which has been shown to be related to a number of variables like external locus of control and educational attainment.

The current study has three strengths: it examined a set of inter-correlated social and psychological factors together determining to what extent each factor influenced the outcome variable; it used a large, nationally representative longitudinal dataset; and it used a set of financial well-being measures (earnings, house ownership status, living space), thus covering more than one components of the concept of financial well-being.

Gender pay gap is well documented [22], though explanations vary. Women in almost every social sectors have less earnings than men. However, the discrepancy of pay between men and women seems to decrease in some developed countries. For example, in the UK, when full-time work is taken in isolation, women earn 10 per cent less than men in 2013. It means the gap between men and women’s full-time earnings has now almost halved since records began in 1997 [22].

There have been numerous studies on the possible causes of the established gender difference in pay which include gender differences in education, hours worked, occupational prestige, employment sector and years in the labour market [23,24,25]. Semykina and Linz (2007) [26] found gender differences in personality traits which explained 8% of the gender wage gap. They also found women’s earnings are strongly affected by personality while the effect of personality on men’s earnings was small and often not significant. Ashby and Schoon (2010) [27] found teenage career aspirations and ambition predicted adult social status and earnings but that the effect was slightly different between the sexes. This study considers the effects of cognitive and non-cognitive individual difference factors separately for males and females. It uses an archived data set that has been used to explore how early childhood factors predict socioeconomic outcomes in adulthood.

### Hypotheses 

This study explored the effects of a set of socio-economic and psychological factors in childhood and adulthood on adult financial well-being, using structural equation modelling and drawing on data collected from a large representative population sample in the UK. Its primary aim was to examine the relative power of individual difference factors measured before adolescence particularly intelligence, over social class, in predicting adult financial success. Specifically it was hypothesised that (H1) Parental social status would be a significant predictors of adult financial well-being; (H2) Childhood intelligence would be a significant predictor of adult financial well-being; (H3) Locus of control would be a significant predictor of adult financial well-being; (H4) Malaise would be a significant predictor of the outcome variable; (H5) Educational achievement and occupational prestige would be significant predictors of the outcome variable.

## 2. Method

### 2.1. Participants

The study draws on a nationally representative cohort study: the 1970 British Cohort Study (BCS70). The study participants were recruited as part of a perinatal mortality survey (BCS70 comprises 16,571 individuals who were born in Great Britain in a week in April 1970 [28]. The following analysis is based on data collected at birth, age 10, age 16, age 30, age 34, and age 38. The analytic sample comprises 4790 cohort members (53 per cent females), for whom complete data were collected at birth and the follow-ups at age 38 years. Analysis of response bias in the cohort data showed that the achieved adult samples did not differ from their target sample across a number of critical variables (social class, parental education and gender), despite a slight under-representation of the most disadvantaged groups [29].

### 2.2. Measures

(1) *Family social background* includes information on parental social class and parental education. Parental social class at birth was measured by the Registrar General’s measure of social class (RGSC). RGSC is defined according to occupational status [30]. Where the father was absent, the social class (RGSC) of the mother’s father was used. RGSC was coded on a 6-point scale: I professional; II managerial/technical; IIIN skilled non-manual; IIIM skilled manual; IV semi-skilled; and V unskilled occupations [31]. Scores were reversed. Parental education is measured by the age parents had left their full-time education.
(2) *Childhood Intelligence* was assessed at age 10 in school using assessed in school, using a modified version of the British Ability Scales (BAS) which can serve as a measure for childhood IQ. The assessment involved the administration of four sub-scales: word definitions and word similarities which were used to measure verbal ability, and recall of digits and matrices which were used to measure non-verbal ability. The alpha for the four measures combined into a total scale was .92.
(3) *Self-esteem* was measured at age 10. Cohort members completed a 12-item Self-esteem Scale (Yes/No) [32,33]. The alpha was .73.
(4) *Locus of Control* was measured at age 16. Cohort members completed a 19-item Locus of Control Scale (Yes/No) [34]. The alpha was .72.
(5) *Malaise Inventory* is a 24-item self-completion instrument, measuring depression, anxiety and psychosomatic illness [35] and it correlates significantly with previously diagnosed and currently treated depression. The alpha was .81.
(6) *Educational Qualifications* was assessed at age 34, participants were asked about their highest academic or vocational qualifications. Responses are coded to the six-point scale of National Vocational Qualifications levels (NVQ) which ranges from ‘none’ to ‘university degree/higher’/equivalent NVQ 5 or 6.
(7) *Occupational Prestige* was measured at age 38. Current or last occupation held by cohort members were coded according to the Registrar General’s Classification of Occupations (RGSC), described above, using a 6-point classification mentioned above.
(8) *Adult Financial Well-being* is a latent variable indicated by weekly income, house ownership status, and living space (number of rooms) measured at age 38. Participants were asked about their current net payment per week (incomes were logged in the following analyses), number of rooms; and their house ownership status (1 = Live rent-free, 2 = Rent it, 3 = Buying with help of mortgage, 4 = Outright own).

## 3. Results

### 3.1. Descriptive Analysis

First, we examined the mean differences of the three financial well-being indicators by sex. ANOVA showed that there was a sex difference on income which indicated women compared to men were less well paid (F(1,4788) = 740.32, *p* < .001). There were no sex differences for number of rooms and home ownership. 

### 3.2. Correlational Analysis

Table 1 shows the correlations of means and SDs of all variables in the study. The three financial well-being indicators were significantly associated with parental social class, maternal and paternal education, childhood intelligence measures, childhood self-esteem, locus of control at teen, malaise, education and occupation (*p* < .01 to *p* < .001). Gender was significantly (*p* < .001) associated with adult earnings. The highest correlations of net income (at 38 years) were educational qualifications measured at 34 years as well as occupational level. However three of the four IQ tests (measured at age 10), as well as self-esteem (measured at age 10) and locus of control (measured at age 16) were significantly correlated with income to confirm the hypotheses. Also as predicted parental education and social class were significantly associated with income.

### 3.3. Structural Equation Modelling

Structural Equation Modelling (SEM) was used to assess the links among family social status, childhood intelligence and self-esteem, locus of control at teen, malaise, educational qualifications and occupational prestige, and current financial well-being. Paths in the models are designed to correspond with the time sequence in which the variables occurred as well as according to the literature in the area. The SEM model testing was carried out using the structural equation modelling program IBM AMOS 22 [36] using maximum likelihood estimation that can be based on incomplete data, known as the full information maximum likelihood (FIML) approach [37].

**Figure 1:**
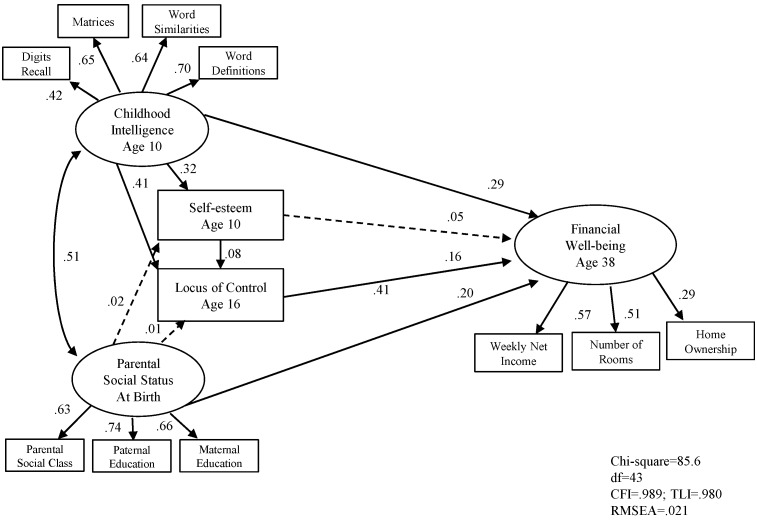
SEM Model 1 early indicators associated with adult financial well-being in male sample (*n* = 2275).

**Figure 2:**
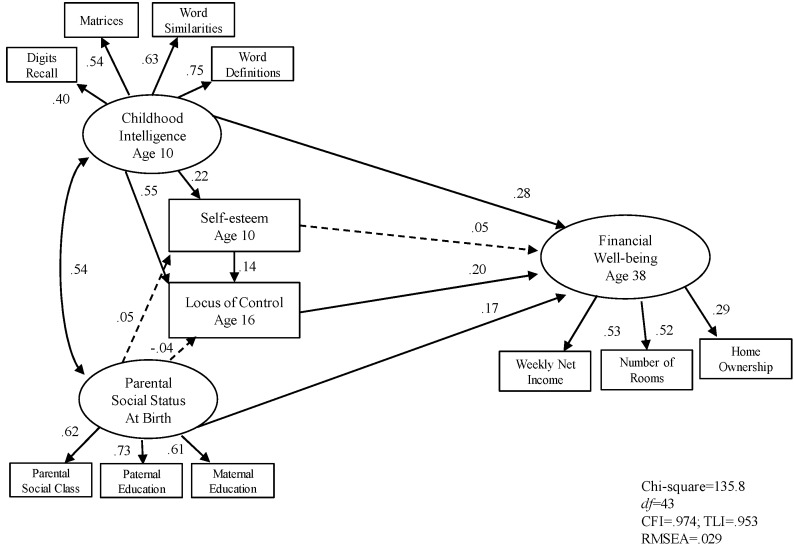
SEM Model 1 early indicators associated with adult financial well-being in female sample (*n* = 2515).

Figure 1 and Figure 2 show the standardised path coefficients of the structural equation models for men and women respectively. The solid lines indicate that the corresponding path coefficients are statistically significant and dashed lines indicate that the path coefficients are non-significant. Error variance for each observable variables and latent variables are included in the model (not shown in the diagrams). 

### 3.4. Model Fit

The χ^2^ statistic is overly sensitive when sample sizes are large or the observed variables are non-normally distributed. The root mean square error of approximation (RMSEA) gives a measure of the discrepancy in fit per degrees of freedom (<.05 indicates a good fit). The final index of choices are the Comparative Fit Index (CFI), and the Tucker Lewis Index (or Non-normed Fit Index) (TLI) where values above .95 indicate a very good fit, and values >.90 are interpreted as good [38]. 

First, we examined the early influences in adult financial well-being. Figure 1 and Figure 2 show the results. Parental social status, childhood intelligence locus of control were all significant predictors of adult financial well-being, accounting for 29 per cent of variance for men and 30 per cent of variance for women respectively.

**Figure 3:**
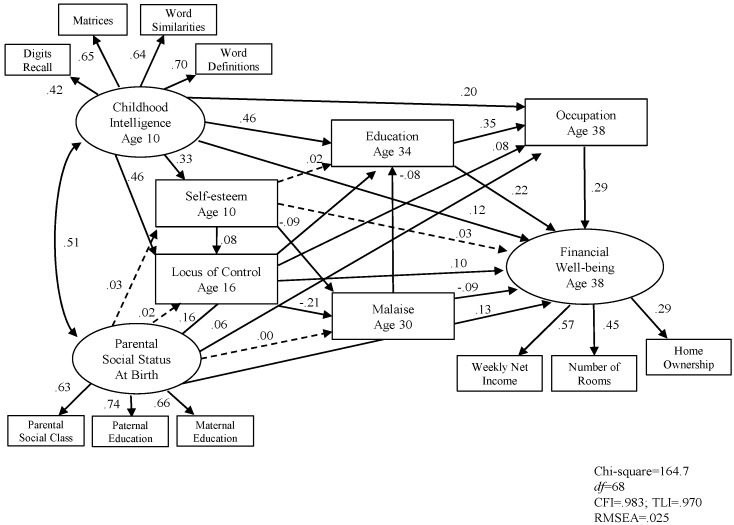
SEM Model 2 predicting adult financial well-being in male sample (*n* = 2275).

**Figure 4:**
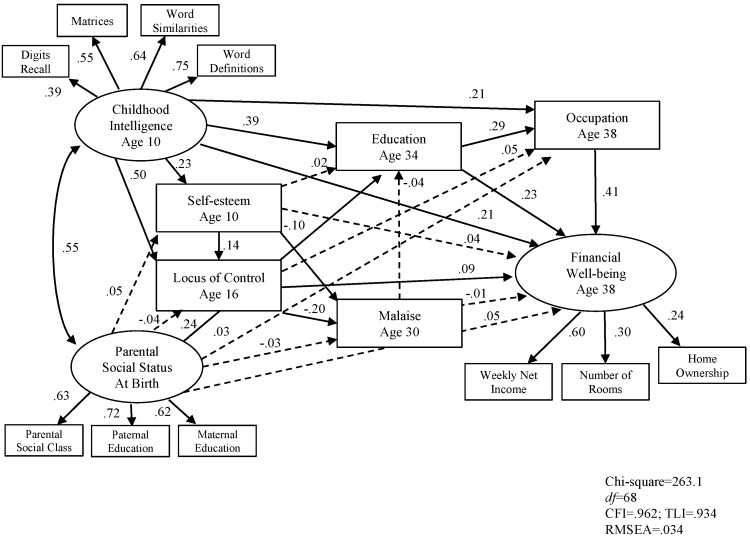
SEM Model 2 predicting adult financial well-being in female sample (*n* = 2515).

Following this, we examined the complete SEM model with education and occupation entered into the model. Results show in Figure 3 and Figure 4. 

Table 2 shows unstandardized estimate, standard error, and standardised estimate of each indicator of the latent variable and the predictors of the outcome variable for the complete SEM model.

The model showed a good fit for men. Chi-square was 164.7 (*df* = 68, *p* < .001), the CFI was .983, the TLI was .970, and the RMSEA was .025. The model explains 45 per cent of the total variance of adult financial well-being. The model also showed a good fit for women. Chi-square was 263.1 (*df* = 68, *p* < .001), the CFI was .962, the TLI was .934, and the RMSEA was .034. The model explains 53 per cent of the total variance of adult financial well-being. Figure 3 and Figure 4 show that for both men and women, childhood intelligence, locus of control, educational qualifications, and occupational prestige were all significantly and independently associated with adult financial well-being. 

For both men and women, the strongest predictor of adult financial well-being was current occupation, followed by education, childhood intelligence, and locus of control at teen.

## 4. Discussion

The results of the current study showed that all of the independent variables measures at different points in time (gender, parental social class and education, intelligence, self-esteem, locus of control, malaise, educational level and occupational status) were all significantly correlated with the financial well-being measure at the item and total score level. Although there were significant differences in total income between the sexes, the pattern of results were strikingly similar.

The results from all the analyses show the extent to which two inter-correlated factors, namely childhood intelligence and parental social class predict financial well-being in mid-life. Whilst this is neither a surprising nor new finding what is interesting is the amount of variance accounted for by these two factors.

Figure 1 and Figure 2 show the results of exploring the possibility of moderator variables. They also show that the direct effect of intelligence is stronger than that of parental social class in predicting adult financial well-being. There are four important points resulting from that analysis. First, that almost a third of the variance could be accounted for by these four factors alone, all of which were measured at least 20 years before mid-life adult well-being. Second, that the pattern for men and women was almost identical. Third, that intelligence and social class both influence self-esteem, but that it is only intelligence that influences locus of control which is directly related to the outcome variable. Fourth, that the strongest relation was between intelligence and locus of control, suggesting that more intelligent children (aged 10) have a more instrumentalist, internal locus of control at age 16 which is a significant predictor of educational success.

Sociologists tend to focus on social class, educational and occupational correlates of financial well-being arguing, as our data indicated, that social class affects educational outcomes and then occupational choices which have a direct influence on financial variables of different kinds. Psychologists on the other hand tend to focus on individual difference correlates and determinants of wealth, showing that personality and intelligence influence educational outcomes which in turn influences financial well-being. There is agreement that education, occupation and financial well-being are highly inter-correlated but more disagreement on the determinants of those variables.

The final results shown in Figure 3 and Figure 4 are interesting for many reasons. The results show that both social class and intelligence predict education which predicts financial well-being. First, the seven variables account for around half the variance for both males and females. Next, for both sexes it is the moderating effect of education that is stronger than parental social class on financial well-being though both are related. Third, for females the role of parental social class is much reduced, compared to males and its effect on financial well-being is moderated by self-esteem, locus of control and education. Fourth, the effect of malaise was weak but in the predicted direction.

It is not difficult to construct a causal theory from these results. High social class parents have higher intelligence which their children partly inherit. Parental socialisation along with school success leads these children to have high self-esteem, an internal locus of control, and a reduced sense of malaise. Intelligence is strongly related to educational outcomes which opens greater and better job opportunities both of which lead to higher salaries.

All studies have their limitations and this is no exception. The sample with complete data had a slight under-representation of lower/manual occupational classes which may provide a small bias in these results. It would have been desirable to have more measures of financial well-being, as well as other psychological (i.e., personality traits) measures to get a more nuanced result. Further, the loadings of the latent outcome variables were relatively low. Therefore results of the current study are not conclusive. Future studies with better psychological properties or more sophisticated identifications of different type of indicators (effect/causal/composite indicators, etc.) and treatment [39] are required to confirm or refute the findings.

The present study confirms the strong and direct effects of parental social class and intelligence on education, occupational and financial well-being in middle age. Current occupation is the strongest predictor of financial well-being, followed by educational achievement, and childhood intelligence is the best predictor of educational attainment. There is a continuous and persistent effect of parental social class on adult financial well-being yet intelligence and locus of control shared a substantial amount of the variance.

## Figures and Tables

**Table 1:** Pearson correlations among gender, parental social status measures, childhood intelligence measures, locus of control, self-esteem, educational qualifications and occupational levels, and adult financial well-being measures.

Variables		Correlation																
		Mean SD	1	2	3	4	5	6	7	8	9	10	11	12	13	14	15	16
1.	Weekly net income	402.5 (307.3)	–															
2.	Number of rooms	5.06 (1.61)	**.209**	–														
3.	House ownership	2.93 (.56)	**.116**	**.203**	–													
4.	Gender	.53 (.50)	**−.366**	**.003**	**.003**	–												
5.	Parental social class	3.37 (1.20)	**.158**	**.057**	**.159**	−.034	–											
6.	Paternal education	15.54 (1.15)	**.147**	**.051**	**.110**	−.011	.460	–										
7.	Maternal education	15.50 (1.05)	**.132**	**.046**	**.119**	−.010	.351	.491	–									
8.	Word Definition scores	11.05 (4.88)	**.246**	**.080**	**.162**	−.133	.296	.257	.262	–								
9.	Word Similarities scores	28.80 (4.06)	**.198**	**.100**	**.132**	−.109	.269	.226	.218	.624	–							
10.	Digits recall scores	22.78 (4.14)	**.095**	**.064**	**.097**	.031	.116	.117	.083	.294	.265	–						
11.	Matrices scores	16.50 (5.09)	**.152**	**.107**	**.148**	.033	.211	.174	.178	.415	.405	.249	–					
12.	Self-esteem	8.79 (2.60)	**.134**	**.101**	**.080**	−.090	.110	.098	.123	.207	.166	.121	.184	–				
13.	Locus of control	14.39 (3.15)	**.177**	**.143**	**.111**	−.012	.165	.143	.134	.320	.283	.166	.259	.216	–			
14.	Malaise	3.09 (3.05)	**−.095**	**−.093**	**−.092**	.095	−.066	−.050	−.066	−.065	−.061	−.058	−.100	−.155	−.210	–		
15.	Educational qualifications	2.68 (1.37)	**.285**	**.195**	**.146**	.032	.313	.272	.284	.390	.323	.183	.341	.181	.335	−.127	–	
16.	Occupational levels	4.21 (1.15)	**.335**	**.173**	**.138**	−.032	.210	.190	.182	.287	.245	.169	.253	.146	.223	−.072	.451	–

Note: Variables were scored such that a higher score indicated being female, more on current income, house ownership, more rooms in the house, a more professional occupation for the parent and higher age parents left school, higher verbal and non-verbal ability test scores in childhood, higher scores on childhood self-esteem, higher scores on locus of control at teen, higher scores on malaise, highest educational qualification, and a more professional occupation.

**Table 2:** Unstandardized estimate, standard error and standardised estimate of the latent and observable variables of SEM that predict adult financial well-being for male and female samples.

Variables	Males			Females		
	Unstandardized Estimate	Standard Error	Standardised Estimate	Unstandardized Estimate	Standard Error	Standardised Estimate
*Parental social status*						
RGSC	1		.633	1		.625
Father’s education	1.153	.053 ***	.762	1.117	.053 ***	.715
Mather’s education	.887	.044 ***	.655	.885	.044 ***	.62
*Childhood Intelligence*						
Word Definition scores	1		.701	1		.747
Word Similarities scores	.734	.028 ***	.64	.735	.030 ***	.635
Digits recall scores	.496	.034 ***	.416	.455	.033 ***	.389
Matrices scores	.962	.047 ***	.654	.795	.044 ***	.555
*Adult financial well-being*						
Weekly net income	1		.574	1		.596
Number of rooms	.004	.001 ***	.448	.003	.001 ***	.296
House ownership	.001	.001 ***	.286	.001	.001 ***	.236
*Predicting adult financial well-being*						
Parental social status	33.174	11.491 **	.134	10.536	9.000 ***	.045
Childhood Intelligence	6.177	3.763 *	.116	8.707	3.530 *	.213
Self-esteem	2.676	2.702	.034	1.906	1.818	.036
Locus of control	6.092	3.617 *	.099	5.295	3.769 *	.085
Malaise	−5.848	2.087 **	−.091	−.502	1.503	−.011
Educational qualifications	29.605	5.638 ***	.217	23.965	3.930 ***	.228
Occupational levels	33.174	5.927 ***	.293	51.180	3.992 ***	.406

Note: * *p* < .05; ** *p* < .01; *** *p* < .001.
